# Longitudinal analysis of tobacco and vape retail density in California

**DOI:** 10.18332/tid/153506

**Published:** 2022-10-17

**Authors:** Vidya Purushothaman, Raphael E. Cuomo, Eric Leas, Jiawei Li, David Strong, Tim K. Mackey

**Affiliations:** 1 Department of Anthropology, University of California San Diego, San Diego, United States; 2 Global Health Policy and Data Institute, San Diego, United States; 3 Department of Anesthesiology, University of California San Diego School of Medicine, San Diego, United States; 4 Herbert Wertheim School of Public Health and Human Longevity Science, University of California San Diego, San Diego, United States; 5 S-3 Research, San Diego, United States

**Keywords:** tobacco, vaping, tobacco retail, longitudinal study, ecological study

## Abstract

**INTRODUCTION:**

Tobacco retailer density may be associated with greater youth initiation and reduced success during quit attempts; however, the extent to which tobacco retailer density has changed overtime across multiple categories of retailers has not been reported.

**METHODS:**

Data on licensed tobacco retailers within California from 2015–2019 were obtained from the California Department of Tax and Fee Administration. Store type was categorized by automated cross-referencing with Yelp. Geolocations were aggregated at county level for analyzing longitudinal trends in changes in tobacco retail density including demographic characteristics.

**RESULTS:**

The number of active CA tobacco retailer licenses increased from 19825 in 2015 to 25635 in 2019. The highest percent increase in tobacco retailer licenses (9.1%) was observed in 2017. The number of specialized tobacco stores was highest in Los Angeles, San Diego, and Riverside counties. We observed a significant increase in the number of active licenses for non-specialized and specialized tobacco stores, both overall and after controlling for the size of populations within each region. Time was a statistically significant predictor for the number of active licenses for only non-specialized stores, after adjusting for covariates. Regional volume of retailers was positively associated with higher proportion of women, lower median household income, and higher proportion of Hispanic residents.

**CONCLUSIONS:**

Monitoring the changes in tobacco retail density and associated sociodemographic factors over time can help to identify communities at higher risk for tobacco and nicotine product exposure and access, and its associated health disparities.

## INTRODUCTION

Greater retail access to tobacco products has been associated with higher smoking rates^1^ among youth^2,3^ as well as adults^4^. Further, retail presence and availability can increase exposure to industry marketing, increase appeal of products potentially leading to greater uptake, and discourage cessation and quit attempts^5,6^. Proximity to tobacco retail outlets has also been associated with higher smoking prevalence^7^, and the number of stores can influence the perception of product availability and ease of access^8^. In an attempt to address known tobacco and nicotine-related health harms, in 2016, the US Food and Drug Administration (FDA) extended its regulatory authority to all tobacco products, including Electronic Nicotine Delivery Systems (ENDS) (‘Deeming Regulation’)^9^. Hence, understanding variation in tobacco retailer density can help in designing targeted tobacco control efforts aimed at reducing appeal, initiation, uptake, and use of these addictive products.

Prior studies have identified several patterns of inequalities in tobacco retail density across various community-level sociodemographic factors, including income, age, and proportion of certain racial minority populations. Tobacco retail density has been found to be associated with smoking among young adults and adolescent smoking^8,10^. Tobacco retail density and smoking prevalence are higher in impoverished areas, even after adjusting for other sociodemographic factors^11^. Multiple studies have also observed a significant association between tobacco retail density and racial/ethnic minority populations in a given community^12,13^. Hence, reducing the tobacco retail density of stores marketing and selling tobacco products may be an effective tobacco control strategy, particularly among populations at higher-risk for tobacco initiation and use^6^.

Understanding the impact of higher concentrations of tobacco retail density engaged in tobacco sales and its relation to initiation and use is particularly important as the tobacco product landscape is evolving, including market-entry of different combustible and non-combustible tobacco and nicotine products (e.g. e-cigarettes, vaping devices, heat-not-burn, etc.). Consumer choices for tobacco products have grown, also leading to a higher proportion of youth and young adults using diverse tobacco products including ENDS due to their convenience, appeal, and effectiveness as nicotine delivery devices that are highly addictive^14,15^. The diversity of tobacco products has also led to an increase in the number of specialized stores that exclusively sell tobacco and vape products, also known as ‘smoke shops’ or ‘vape shops’ or ‘head shops’. These changes mean that identifying differential patterns in tobacco retail density growth, based on store type and predominant product sold, is needed to assess the impact on tobacco product exposure and use patterns.

In California, every retailer who sells cigarettes or tobacco products to the public is required to obtain a cigarette and tobacco retailer’s license from the California Department of Tax and Fee Administration (CDTFA) and renew it annually^16^, in accordance with the California Cigarette and Tobacco Products Licensing Act of 2003. In June 2016, coinciding with the FDA’s Deeming Regulations, state law expanded the definition of a tobacco product to include: ‘Any product containing, made, or derived from tobacco or nicotine that is intended for human consumption, any electronic smoking or vaping device that delivers nicotine or other vaporized liquids, any component, part, or accessory of a tobacco product, whether or not sold separately’, effectively extending the license requirements to retailers selling ENDS and other emerging nicotine products^16^.

As of 10 November 2020, over 22000 retailers were licensed to sell tobacco products in the state of California (approximately 56 tobacco stores per 100000 population)^17^, excluding individual retailers (sole proprietors, husband and wife co-owners, and domestic partners), wholesalers and distributors. This large number of licensed tobacco retailers reflects a retail landscape that has expanded to include specialized stores, including specialized smoke shops and vape shops that cater to enthusiasts who prefer new and diverse types of tobacco products^18^. While these specialized stores often offer a greater range of tobacco products, non-specialized stores such as convenience stores or grocery stores may be more accessible point-of-sale locations for the typical smoker. However, previous research has also observed that neighborhoods with high numbers of young adults had higher numbers of specialized tobacco/vape retailers, which may indicate a potential for increased risk of tobacco uptake and use^19^.

In general, tobacco outlets can be broadly categorized into: 1) specialized stores (vape-specific stores such as vape shops, tobacco-specific stores such as smoke shops); and 2) non-specialized stores (grocery stores, gas stations, convenience stores). Though the CDFTA list of licensed retailers does not distinguish between the types of tobacco retail categories, other online sources, such as the crowdsourced business listing website Yelp^20^, provide this information for consumers as part of online business listings and directories. Irrespective of the retail store type, the tobacco industry spends most of their total marketing expenditure at the point-of-sale in the retail environment. In 2019, the industry spent over 81% of their total marketing expenditures on point-of-sale marketing in the retail environment^21^.

While some studies have examined the association between tobacco retail density and sociodemographic factors, only a few studies have used Yelp to classify tobacco outlets as specialized tobacco stores (e.g. smoke shops, vape shops) and non-specialized tobacco stores (e.g. convenience stores, grocery stores etc.)^22,23^. This study aims to use combined state licensure data and business listings to identify and characterize specialized tobacco stores, their location, marketing exposure and access, in comparison with non-specialized tobacco stores. This study aims to assess longitudinal trends in the number of specialized and non-specialized tobacco stores along with their associated sociodemographic factors at the county level in the state of California.

## METHODS

### Data collection

A list of licensed tobacco retailers from 1 January 2015 to 31 December 2019 was obtained in the month of June 2020 from the California Department of Tax and Fee Administration (CDTFA)^17^. However, this list did not include retailers registered as individuals (sole proprietors, husband and wife co-owners, and domestic partners).

The licensed tobacco retailer list includes: 1) license number, 2) owner name, 3) business name, 4) retailer address, 5) date of license commencement, and 6) date of license expiry. In June 2020, store categories listed on Yelp were data mined, based on store names and addresses obtained from CDTFA retail license data using scripts written in the programming language Python (version 3.7.0, Selenium package). The store characteristics data mined were then used to classify the retailers based on Yelp categories (businesses on Yelp are categorized based on feedback from Yelp users, data curation teams as well as owners who claim the store on Yelp)^24,25^ into: 1) specialized stores (labelled as tobacco shops and/or vape shops on yelp that sell various tobacco products including ENDS, cigars, hookah products); and 2) non-specialized stores (e.g. convenience stores, grocery stores, pharmacies, dollar stores etc.). Tobacco retailers which were classified as specialized stores were then further classified into: 1) tobacco specific stores (categorized as tobacco store only); 2) vape specific stores (categorized as vape store only); and 3) tobacco and vape stores (categorized as tobacco store and vape store).

Data for population, age, gender, race, ethnicity, and median household income for each of the years in the period 2015–2019, were obtained from the American Community Survey at the county level for the state of California^26^.

### Data analysis

A total of 31251 retailer licenses were provided by the CDTFA for the years 2015–2019 out of which 26371 (84.4%) licenses were cross-referenced based on the store name and address of the CDTFA retailer license. The 4880 (15.6%) licenses that could not be crossreferenced for store categorization using Yelp were excluded from the analyses. Of the excluded licenses, 42.9% (n=2091) were located in the three most populous counties in California: 28.7% (n=1401) were in Los Angeles County, 8.3% (n=404) were in Orange County, and 5.9% (n=286) were in San Diego County; which was similar to the distribution of the licenses with store categorization (43.6%). It is possible that unmatched licensed businesses represent those that do not register or claim a Yelp listing, are businesses that have shut down or are no longer in operation, or failed to match due to a change of address or incorrect licensure details. The 26371 licenses that were cross-referenced and matched for store categorization using Yelp were used for all the analyses in this study.

Based on the CDTFA license commencement and expiration dates, the total number of active licenses and new licenses issued in every store category were identified for each year in the period 2015–2019, and only accounted for the active years in our analyses. Using the latitude and longitude coordinates for each of the retailer addresses, point coordinates were plotted on a California base map for tobacco retail density in corresponding counties. By aggregating point coordinates, the number of retailers for each county, for each store category, was obtained for each of the years in the period 2015–2019. As the data on tobacco retailer count at sub-county geographies exhibited limited variability across geospatial units, examining the longitudinal trends and associations at the county level enabled better continuous gradients and was more appropriate for linear analysis.

The numbers of active licenses and new licenses for each store category for each year were calculated. A mixed-effects linear regression model was used to determine if the retailer count significantly changed over time (2015–2019) using a random effect to account for repeated sampling of each county and modelled by Gaussian error distributions. Separate models were run for non-specialized and specialized tobacco stores using the retailer count by store type for each year as the dependent variable. County population for each year was included in all models to offset store counts. Further, the model was adjusted for other sociodemographic covariates such as county-level population count by gender, age, race/ethnicity groups, and annual household income for each year. All the dependent and independent variables were treated as continuous variables. Independent variables were divided by 100000 to scale up effect estimates. The variance inflation factor (VIF) was assessed to analyze multicollinearity of model terms. All statistical analyses were conducted using SPSS version 27. A p<0.05 was considered statistically significant.

Spatial clusters of high values (hot spots) and low values (cold spots) for specialized tobacco and/or vape retailer stores adjusted for population were mapped using the optimized hot-spot analysis tool in ArcGIS v10.7.1 (Esri: Redlands, CA). This tool calculates the Getis-Ord Gi* statistic and allows for the mapping of related z-scores. The median household income by county was overlayed using proportional symbology using natural breaks that best group similar values and maximize the differences between classes. Overlaying median household income over the map of hot-spot analyses can help in examining the sociospatial patterning of retail outlets^27^. Geographically weighted regression was used to calculate model AIC and R^2^ to verify spatial model fit.

## RESULTS

The number of active licenses during a given year increased from 19825 in 2015 to 25635 in 2019. The highest percent increase in tobacco retailer licenses (9.1%) was observed in 2017. The highest percent increase in active licenses for tobacco-specific (13.8%) and vape-specific stores (105%) were both observed in the year 2016. A total of 8306 new licenses were issued from 2015 to 2019. The highest number of new licenses (2226) was issued in the year 2017 (Table 1). While the increase in the number of specialized retailers increased by 26.5% in each year during 2016–2017, increase in vape-specific stores accounted for 41.7% (n=105) and 50.5% (n=161) of the increase in overall specialized stores in 2016 and 2017, respectively.

**Table 1:** Number of active and new tobacco retailer licenses that are specialized and non-specialized stores (2015–2019)

*License category*	*Year*	*Specialized stores*			*Non-specialized stores n (% change)*	*Total n (% change)*
		*Overall n (% change)*	*Tobacco specific n (% change)*	*Vape specific n (% change)*		
**Active licenses**	2015*	950	585	100	17925	19825
	2016	1202 (26.5)	666 (13.8)	205 (105.0)	18757 (4.6)	21161 (6.7)
	2017	1521 (26.5)	747 (12.2)	366 (78.5)	20044 (6.9)	23086 (9.1)
	2018	1827 (20.1)	842 (12.7)	452 (23.5)	21249 (6.0)	24903 (7.9)
	2019	2054 (12.4)	907 (7.7)	496 (9.7)	21527 (1.3)	25635 (2.9)
**New licenses**	2015*	183	95	18	1474	1840
	2016	268 (46.45)	86 (-9.47)	109 (505.6)	1550 (5.16)	2086 (13.37)
	2017	332 (23.88)	88 (2.33)	161 (47.71)	1562 (0.77)	2226 (6.71)
	2018	340 (2.41)	112 (27.27)	97 (-39.75)	1675 (7.23)	2355 (5.80)
	2019	305 (-10.29)	98 (-12.50)	63 (-35.05)	1085 (-35.2)	1695 (-28.03)

* Percent change not included for the year 2015.

Mixed effects linear regression modeling demonstrated a significant change from 2015 to 2019, during which the number of active licenses for all store categories increased with time (p<0.05), after adjusting for differences in county populations (Table 2). The highest effect estimate was observed among non-specialized stores (fixed effects estimate= 17.94 ± 5.81, p=0.003; variance p<0.001). For the number of new licenses issued, a trend towards significant increase over time was observed for specialized tobacco and/or vape stores (fixed effects estimate=0.52 ± 0.28, p=0.07; variance p<0.001), with non-specialized tobacco vendors showing a significant decreasing trend over time (fixed effects estimate= -1.26 ± 0.48, p=0.009; variance p=0.01) (Table 2).

**Table 2:** Mixed effects linear regression models by store type for active and new tobacco retail licenses (2015–2019)

*Specialized stores (active licenses)*						
*Fixed effects*				*Variance (county)*		
*Effect*	*Estimate*	*SE*	*p*	*Estimate*	*SE*	*p*
Year	5.05	1.85	0.008	13.87	4.06	0.001
Population	1.52	0.04	<0.001			
***Vape-specific stores (active licenses)***						
***Fixed effects***				***Variance (county)***		
***Effect***	***Estimate***	***SE***	***p***	***Estimate***	***SE***	***p***
Year	1.84	0.63	**0.005**	22.89	4.34	**<0.001**
Population	0.01	0.02	0.52			
***Tobacco-specific stores (active licenses)***						
***Fixed effects***				***Variance (county)***		
***Effect***	***Estimate***	***SE***	***p***	***Estimate***	***SE***	***p***
Year	1.46	0.54	**0.009**	11.13	3.22	**<0.001**
Population	1.23	0.03	**<0.001**			
***Non-specialized stores (active licenses)***						
***Fixed effects***				***Variance (county)***		
***Effect***	***Estimate***	***SE***	***p***	***Estimate***	***SE***	***p***
Year	17.94	5.81	**0.003**	1719.58	335.91	**<0.001**
Population	42.31	0.38	**<0.001**			
***Specialized stores (new licenses)***						
***Fixed effects***				***Variance (county)***		
***Effect***	***Estimate***	***SE***	***p***	***Estimate***	***SE***	***p***
Year	0.52	0.28	0.07	3.73	0.86	**<0.001**
Population	0.53	0.02	**<0.001**			
***Vape-specific stores (new licenses)***						
***Fixed effects***				***Variance (county)***		
***Effect***	***Estimate***	***SE***	***p***	***Estimate***	***SE***	***p***
Year	0.12	0.11	0.29	-	-	-
Population	0.27	0.01	**<0.001**			
***Tobacco-specific stores (new licenses)***						
***Fixed effects***				***Variance (county)***		
***Effect***	***Estimate***	***SE***	***p***	***Estimate***	***SE***	***p***
Year	0.04	0.07	0.60	<0.001	0.01	0.94
Population	0.31	0.01	**<0.001**			
***Non-specialized stores (new licenses)***						
***Fixed effects***				***Variance (county)***		
***Effect***	***Estimate***	***SE***	***p***	***Estimate***	***SE***	***p***
Year	-1.26	0.48	**0.009**	25.98	10.31	**0.01**
Population	3.96	0.07	**<0.001**			

Population divided by 100000 to scale up effect estimates.

After controlling for sociodemographic covariates (age, gender, race, ethnicity, median household income, county population), the longitudinal increase retained statistical significance only for the number of active licenses for non-specialized tobacco retailer stores (p=0.003). For specialized tobacco and/or vape stores, the longitudinal increase was no longer statistically significant after adjusting for covariates, although it exhibited a trend towards significance (p=0.09). Other statistically significant covariates are listed in Table 3. The independent variables were divided by 100000 to scale up effect estimates. The spatial model fit for the adjusted model was verified using Geographically Weighted Regression (AIC=602.66, R^2^=0.67).

**Table 3:** Mixed effects linear regression model for active tobacco retailer licenses adjusting for covariates (2015–2019)

*Model*	*Parameter*	*Specialized tobacco and/or vape stores*			*Non-specialized stores*		
		*Estimate*	*SE*	*p*	*Estimate*	*SE*	*p*
**Random effect**	Variance	164.28	45.72	**<0.001**	1914.60	619.72	**0.002**
**Fixed effects**	Intercept	11.35	6.91	0.11	33.75	22.49	0.14
	Year	0.81	0.47	0.09	4.43	1.46	**0.003**
	Population	-9.52	5.71	0.11	-26.87	19.30	0.18
	Male population	57.68	29.82	0.06	152.00	98.01	0.13
	Female population	108.57	25.78	**<0.001**	374.40	85.18	**<0.001**
	15–19 years population	-286.04	59.58	**<0.001**	-1161.44	187.43	**<0.001**
	20–24 years population	-181.63	33.68	**<0.001**	-333.14	108.02	**0.003**
	25–34 years population	21.75	31.53	0.49	36.74	100.64	0.72
	35–44 years population	-277.95	32.58	**<0.001**	-434.13	101.36	**<0.001**
	45–54 years population	-86.00	38.01	**0.03**	-436.54	120.11	**<0.001**
	White population	7.15	5.36	0.18	12.79	16.68	0.44
	African American population	3.54	12.92	0.78	-55.37	41.50	0.19
	Asian population	11.87	8.15	0.15	2.25	25.68	0.93
	AIAN* population	-96.50	93.75	0.31	901.36	294.98	**0.003**
	NHPI** population	-147.99	122.2	0.23	-388.52	405.20	0.34
	Hispanic population	11.10	4.31	**0.01**	49.14	14.05	**0.001**
	Median household income ($)	-36.91	12.83	**0.005**	-99.30	41.74	**0.02**

#Independent variables were divided by 100000 to scale up effect estimates. All independent variables are expressed as count except median household income which is expressed in US dollars.

* American Indian/Alaska Native.

** Native Hawaiian/Pacific Islander.

While Hispanic population had a significant positive association with retail quantity for both nonspecialized and specialized tobacco retail density, American Indian/Alaska Native (AIAN) population only exhibited a significant positive association with the number of non-specialized retailers. Also, population of people aged 35–44 years had a significant negative association with the number of specialized (-277.95 ± 32.58, p<0.001) as well as non-specialized stores (-434.13 ± 101.36, p<0.001). This indicates that the average number of non-specialized stores was lower by 2.77 and specialized stores was lower by 4.34 for every 1000 increase in population of people aged 35–44 years, for a given median household income and population distribution by gender and race/ethnicity. Median household income had a significant inverse association with retailer quantity of both non-specialized (-99.30 ± 41.74, p=0.02) as well as specialized tobacco stores (-36.91 ± 12.83, p=0.005). This indicated that the average number of non-specialized stores was higher by 9.93 and specialized stores was higher by 3.69 for every $10000 decrease in median household income, for a given population distribution by age, gender, and race/ethnicity. The VIFs for median household income were 1.53 (specialized tobacco stores model) and 1.38 (non-specialized stores model). The VIFs for the other predictors (population by gender, age, race/ethnicity) ranged 4.89–6.02.

**Figure 1:**
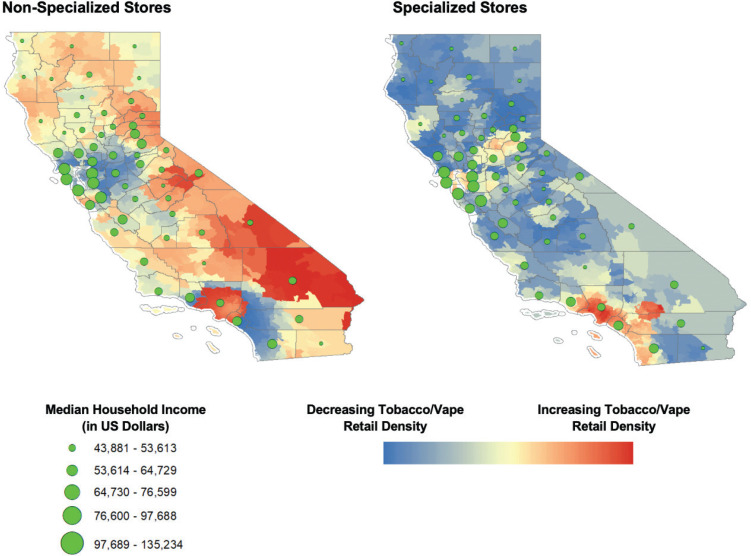
Z-scores for spatial hot spots of tobacco retail density adjusted for population in California with overlay of median household income at county level (2019)

Additionally, in 2019, the tobacco retail density of non-specialized stores in Trinity County (lowest median household income) was 1.7 per 1000 residents compared to 0.5 per 1000 residents in Santa Clara County (highest median household income). For the year 2019, a visualization of z-scores across California with an overlay of median household income revealed clustering of high z-scores (hot spots) for specialized tobacco and/or vape retail density (adjusted for population) in densely populated counties such as Los Angeles County, Orange County, and San Diego County (Figure 1).

## DISCUSSION

Using detailed business listing data from Yelp, this study cross-referenced and categorized over 25000 California licensed tobacco retailers according to the types of tobacco and ENDS products that they sold to the public. Overall, this study found an increasing trend in the number and density of tobacco retailers from 2015 to 2019. While we observed fluctuations in the annual percent change of the number of licenses, the overall number of active licenses increased for both specialized and non-specialized tobacco retailers reflecting overall growth of the industry despite the passage of several progressive tobacco control policies in the state during 2015–2019 (e.g. Proposition 56).

The highest increase in the total number of new licenses was observed from 2015 to 2016 and the highest increase in the total number of active licenses was observed from 2016 to 2017, which conforms to the state’s legal expansion of the definition of tobacco products in June 2016 to include ENDS^16^. While the trend of change in new tobacco licenses may reflect an increase in licensure activity for retailers selling ENDS products (which became subject to tobacco retail licensure requirements and are treated as tobacco products), the trend of change in the active licenses also takes into account the possibility of expired licenses or stores that might have gone out of business. Reflecting this effect, the highest increase in the number of vape-specific stores was observed in 2016 (505%), which is again consistent with the expanded licensure definition to ENDS. It is important to note that, while a substantial proportion of these vape-specific stores could have already existed prior to the licensure requirement taking effect, the study nevertheless observed a significant increase in the number of vape-specific stores even after 2016, including a substantial increase in 2017 indicating that popularity of these stores may have increased irrespective of licensure requirements.

The second key finding of this study related to the above was the observation of a significant longitudinal increase in the number of active licenses issued to specialized tobacco and/or vape retailers after adjusting for county population. Increasing numbers of specialized tobacco and/or vape stores may influence availability and appeal specific to ENDS products, leading to greater uptake, use, and associated addiction to these specialized nicotine delivery devices. Prior research has shown that living in neighborhoods with higher tobacco retail density has been associated with higher odds of initiating alternative tobacco use among adolescents^10^. Specifically, retail store tobacco advertising has been shown as a risk factor for smoking initiation among adolescents^28^. This tobacco retail risk environment also includes the potential impact of non-specialty stores, where the odds of tobacco use initiation increased with the frequency of visiting convenience and grocery stores that were actively advertising cigarette products^28^.

Thirdly, median household income was inversely associated with both the number of specialized tobacco/vape stores as well as non-specialized stores in the adjusted longitudinal model. This finding is consistent with the existing literature on socioeconomic disparities associated with tobacco retail density^29^. Previous studies have observed disproportionate distributions of tobacco retail outlets within socioeconomically disadvantaged neighborhoods^2,13^. For example, a prior study observed that implementing a ban on the sale of tobacco products within 1000 feet of schools in New York and Missouri could potentially reduce tobacco use disparities associated with income and the proportion of the African American Population^30^. This study similarly observed a significant association between low-income communities and retail density of specialized tobacco and/or vape stores that requires further sub-regional and community-specific analyses.

Further, prior studies have found that tobacco retail density was positively associated with the proportion of Hispanic and African American people in communities^31^. Our study also found positive associations with tobacco retail density and Hispanic populations for both non-specialized and specialized tobacco stores, with American Indian/Alaska Native (AIAN) populations also exhibiting a significant positive association for non-specialized retailers, reflecting ongoing trends in specific racial tobacco disparities in the state among two priority population groups. As tobacco-related health disparities continue to be perpetuated by a growing tobacco retail product landscape targeting these communities, licensing policies may represent a useful tobacco control tool in preventing the clustering of tobacco retail outlets in these higher risk communities if they are more progressively enforced to limit the density of tobacco retailers in certain areas or denying licenses/permits.

Finally, this study also observed an inverse association between tobacco retail density (both specialized and non-specialized) and proportion of youth and young adults in California counties. However, despite this inverse trend, prior literature suggests that any tobacco retail density can influence smoking rates among youth and young adults^3^. Youth with easy access to convenience stores that sell e-cigarettes and exposure to marketing advertisements have been observed to have higher risk of e-cigarette initiation^32^. Studies have also observed that price promotions on tobacco products were associated with higher youth smoking prevalence^33-35^. Such price promotions in areas with lower median household income can induce a sense of easy accessibility and make tobacco use more appealing. Retail licensing policies should aim at further reducing the clustering of tobacco/vape-specialized stores in socioeconomically marginalized neighborhoods to reduce existing disparities while also coupling these efforts with more active restriction of tobacco/ENDS point-of-sale marketing and sales promotions aimed at inducing tobacco initiation from retail sources.

### Implications

We identified a significant longitudinal increase in active licenses for specialized and non-specialized tobacco retail stores adjusted for population and the trend remained significant for non-specialized stores after adjusting for relevant sociodemographic factors. Additionally, sociodemographic factors such as lower median household income, higher proportion of minority population (Hispanic residents) and higher percentage of female population were associated with greater increase in the county level retailer count of non-specialized tobacco retailers over time. Clustering of tobacco retail outlets in low-income and minority neighborhoods can lead to health disparities associated with tobacco use.

### Limitations

This study provides preliminary evidence of increasing numbers of tobacco retailers in the state of California, including statistically significant differences in licensing trends between specialized and non-specialized tobacco stores. However, this study has some limitations. The store categorization was based on publicly available Yelp data and certain limitations inherent to such websites can extend to the study. One such limitation is the ability for businesses to self-classify^36^ which may lead to misclassification bias. Additionally, the CDTFA retailer listing obtained does not include individuals (sole proprietors, husband and wife co-owners, and domestic partners) who are registered with, or hold licenses or permits issued by, the California Department of Tax and Fee Administration, due to privacy issues. This data gap is important as a considerable number of vape stores are run by single-store owners^37^ or are small businesses^38^. Hence, this study was not able to capture the change in trends for individually owned licensed tobacco retailer density owing to the privacy restrictions which may have led to bias in the dataset due to oversampling of larger and incorporated businesses. Further, although Yelp is a lucrative way to increase visibility of businesses, it requires business owners to create a profile to claim their business and spend on additional features to manage the profile which may not be a viable option for all operators. The study did not include 4880 stores (15.6%) which could not be cross-referenced using Yelp, or where Yelp classification of retailer type could not be verified. This could possibly underestimate the county-level retailer count of stores selling tobacco products, irrespective of store type. However, nearly half of the excluded stores were located in the three most populous counties of California, similar to the distribution of the licenses that were included in the analyses. Additionally, the retrospective data on licensed retailers from 2015–2019 were mined for store-categorization using Yelp in June 2020, which may not reflect prior categorizations or changes made to a listing. Also, different retailer store types such as convenience stores, gas stations, grocery shops etc., were grouped together as non-specialized stores selling tobacco products since some of the stores in this category could have multiple labels on Yelp. Hence, the study was not able to identify if a sub-category of these non-specialized stores selling tobacco products was significantly higher than the other. The point-of-sale marketing exposure was not measured explicitly and instead used tobacco retail density of specialized or non-specialized tobacco stores as a proxy for exposure. The study also did not specifically assess the effect of the state regulatory change to include ENDS as tobacco products and if there was a lag between policy implementation and licensure registration effect. Future research should explore the effects of this policy change in detail using a pre- and post-implementation study design. Finally, this study analysis was conducted at the state county level since majority of the census tracts had 1 store or no store, thereby limiting the ability of analysis on census tracts to discover linear associations between county-level characteristics and geospatially aggregated store count. The store count was therefore aggregated to the county level to improve variability in the data. The county-level results may not be generalizable to all communities within counties, as each community may have a distinct tobacco retail landscape and community of users. The point data on store locations were aggregated to county-level retailer count, which can lead to modifiable areal unit problem (MAUP) as the results for the same region can be inconsistent based on shape and scale chosen for analysis. Also, the interpretations of the demographic analyses are ecological and do not apply to individual tobacco users.

## CONCLUSIONS

Examining the longitudinal trends of licensed tobacco sellers allows for valuable insights when monitoring growth of the increasingly diversified tobacco retail industry landscape. Further, examining the sociodemographic factors that are associated with communities that have a higher burden of tobacco retail density exposes the disproportionate distribution of these access points that can worsen existing health disparities caused by tobacco use and nicotine addiction. New licensing policies aimed at better regulating retail growth and density, particularly in the context of areas already with high levels of retail establishment exposure or whose populations are at heightened risk for tobacco uptake, may help combat continued tobacco-related health disparities within these communities. More targeted and effective tobacco control policies may be shaped by the specific needs of communities that have to deal with the consequences of tobacco addiction. Future research should seek to further evaluate the potential for more progressive tobacco retailer licensing policies to enable reductions of tobacco-related disparities specifically associated with youth, racial/ethnic minorities and lower income individuals in the state of California and in other jurisdictions.

## Data Availability

The data supporting this research are available from the authors on reasonable request.
